# Does Serum lipid profile differ in anemia and non-anemic older subjects?

**DOI:** 10.22088/cjim.8.4.305

**Published:** 2017

**Authors:** Mahshid Shirvani, Mohsen Vakili Sadeghi, Seyed Reza Hosseini, Ali Bijani, Reza Ghadimi

**Affiliations:** 1 Student Research Committee, Babol University of Medical Sciences, Babol. Iran.; 2 Cancer Research Center, Health Research Institute, Babol University of Medical Sciences, Babol, Iran.; 3 Social Determinants of Health (SDH) Research Center, Health Research Institute, Babol University of Medical Science, Babol, Iran.; 4 Infectious Diseases and Tropical Medicine Research Center, Health Research Institute, Babol University of Medical Sciences, Babol, Iran.

**Keywords:** Elderly, Anemia, Hemoglobin, Iron Deficiency Anemia, Lipid Profile, Cholesterol, Triglyceride

## Abstract

**Background:**

There is some evidence that shows the symptoms of anemia are fewere in overweight and obese people, so, the purpose of this research was to study the relationship between anemia and iron deficiency anemia (IDA) with lipid profile status of the elderly.

**Methods:**

This cross-sectional study came from the Amirkola Health and Ageing Project (AHAP). A demographic questionnaire was given to the older people and a blood sample was obtained to assay their lipid indexes (triglyceride, cholesterol, HDL and LDL) and the parameters related to anemia after 12 hours fasting. The data were analyzed by chi-square test, t-test and Pearson correlation using SPSS. A p<0.05 was considered as the significance level of the tests.

**Results:**

The average age of the people was 68.95±7.43 years old. In this study, the prevalence of anemia and IDA was 31% and 9%, respectively. The mean concentration of serum triglyceride, cholesterol and LDL in the anemia group and the IDA group was less than the control groups. The amount of HDL in different groups was almost the same, although the difference was statistically significant with respect to variables like age and obesity (P=0.001).

**Conclusion:**

The study showed that the amount of lipid profile (triglyceride and cholesterol) in the elderly with anemia and IDA was less compared to other people. This result was achieved in some other research studies too, but further research is suggested to find possible mechanisms.

**A**nemia is a common blood disorder as 26% of adults in the developing countries are anemic (1). In Iran, 30% of people aged 3-65 are suffering from anemia (2). Symptoms of moderate anemia include fatigue, loss of energy, shortness of breath, and palpitations (especially during physical activity) (1). One of the most important types of anemia is iron deficiency anemia (IDA) (3). Iron is an essential element in the physiology of the body, such as the transport of oxygen and enzymatic reactions. However, excess iron, due to production of free radicals, is harmful and causes tissue damage (4, 5). Iron deficiency reduces the serum level of iron and ferritin and thereby causes impaired erythropoiesis (6). About 50% of anemia cases in the world can be attributed to iron deficiency which is responsible for about 841,000 deaths in the world every year (1). A study conducted in Iran in 2001 showed that anemia and iron deficiency occursin a high percentage of the population in different age groups. Accordingly, about 23% of children under 2, 26% of children aged 6, 23% of male and female adolescents, and 43% of pregnant women suffer from iron deficiency (7). In a study conducted in the US, it was reported that nearly 4.7 million Americans suffer from anemia and its prevalence increases with age, as 44.4% of people aged over 85 are anemic (8). 

Elevation of serum lipid levels increases the risk of atherosclerosis and coronary heart disease (4). Because of the role of abnormal lipid levels in atherogenesis, its effects on health increase with age, thus great attention is paid to abnormal levels of lipids and its associated factors (9, 10). The results of some studies show that the symptoms of anemia are fewer in overweight or obese people than those with normal weight (11, 12). On the other hand, the findings of another study indicate that anemia is associated with increased risk of long-term complications of cardiovascular events and death, especially in obese patients (13). 

In a number of epidemiological studies, high iron reserves in the body have been reported to be associated with increased risk of coronary heart disease, the most important risk factor is hyperlipidemia (14, 15). In this regard, although the relationship between iron intake and serum lipid level has been found in animal models, this relationship has not been studied extensively in humans (16-18). Since few studies have been conducted on the effect of anemia on serum lipid profile, the present research aimed to study the relationship between lipid profile and IDA. 

## Methods

The present cross-sectional research was part of a cohort study conducted on 1616 elderly people aged 60 or above in Amirkola, Babol, Mazandaran Province (19). Project protocol was approved by the Babol University of Medical Sciences Research Ethics Committee, and a written consent was obtained from all subjects after briefing them on the research objectives. Patients with chronic renal failure along with anemia and blood cancer or the elderly whose information on their serum lipid level and anemia indices was incomplete or unavailable were excluded from the study. Then, the subjects were asked to fill out a questionnaire containing the demographic information. Fasting blood samples were collected and biochemical markers were measured according to the standard method in the laboratory of Cellular and Molecular Biology Research Center, Babol University of Medical Sciences. Lipid indices tests (triglyceride, cholesterol, HDL, and LDL) were conducted based on ELISA method using the kits manufactured by Pars Azmoon Company. 

Parameters related to blood cell count and anemia were studied using Sysmex XS-1000i. TIBC and serum iron levels were measured based on ELISA kits manufactured by Pars Azmoon Company. In addition, ELISA kits of DiaMetra Company were used for the measurement of ferritin. Hemoglobin level less than 14 gr/dl for men and less than 12 gr/dl for adult women, diagnosed anemia cases by the physician and cases under treatment were defined as anemia (1). Patients with a ferritin level of less than 20 µg/dl, transferrin saturation less than or equal to 10%, and serum iron of less than 30 µg/dl and the patients for whom iron supplement was prescribed by the physician were regarded as the cases of IDA (1). 

It is noteworthy that the patients receiving anti-lipid drugs were excluded from the comparison of serum lipid level in anemia and IDA groups. The obtained data and information were statistically analyzed using chi-square, t-test, and correlation coefficient test in SPSS software. The level of significance in this study was determined to be p<0.05.

## Results

Among the 1616 elderly people studied, 22 (1.4%) individuals people were excluded from the study according to the predetermined criteria. Finally, 868 men and 726 women with the mean age of 68.95±7.43 were selected as the subjects. Table 1 shows the demographic information, status of anemia indices, and lipid profile of subjects.

**Table 1:** Demographic information, status of anemia indices, and lipid profile of subjects

**Women**	**Men**	**Variable**
68.24±7.18	69.38±7.82	Age (year)
28.23±4.16	26.03±4.65	BMI (Kg/m^2^)
12.02±1.11	14.17±1.08	Hemoglobin (mg/dL)
83.11±10.12	84.31±9.12	MCV
78.23±32.63	87.15±37.09	Serum iron (µg/dL)
154.21±117.54	167.13±123.61	Ferritin (µg/L)
285.16±36.12	280.12±38.24	TIBC (µg/dL)
173.81±91.19	147.21±77.20	Triglyceride (mg/dL)
211.34±42.08	191.16±37.5	Cholesterol (mg/dL)
39.38±4.12	38.06±4.39	HDL (mg/dL)
144.95±46.32	126.81±39.28	LDL (mg/dL)

Among the subjects, 300 people were taking anti-lipid drugs and 58 people were receiving iron supplement, which was taken into account in data analysis. Among the subjects, 497 (31%) elderly were anemic, with a frequency of 23.8% and 37.3% among among women and men, respectively (P<0.001). Moreover, 157 (9%) subjects had IDA, with a prevalence of 11.9% among females, and 8.2% among males (P<0.001). Table 2 presents the mean serum level of triglyceride, cholesterol, HDL, and LDL in two groups of anemic and non-anemic. After excluding the subjects who were taking anti-lipid drugs, the mean level of triglyceride in anemic and non-anemic groups was 141.98 mg/dl and 166.11 mg/dl, respectively. 

**Table 2:** Mean serum lipid profile in anemic (404) and non-anemic (894) elderly groups

**Pvalue**	**Non anemic** **(Mean±SD)**	**Anemic** **(Mean±SD)**	**Variable**
0.001	166.11±87.23	141.94±76.85	Triglyceride(mg/dL)
0.001	206.02±40.38	187.43±39.25	Cholesterol (mg/dL)
0.23	38.93±4.29	38.60±4.55	HDL (mg/dL)
0.001	139.43±44.95	121.89±36.54	LDL (mg/dL)

The difference between these two groups was statistically significant in this regard (p=0.001). Moreover, serum cholesterol level in anemic group (187.43 mg/dl) was significantly lower than the control (206.02 mg/dl). The mean HDL was almost the same in anemic and non-anemic groups (P=0.23), while the mean LDL in the anemic group was significantly lower than the non-anemic group (P=0.001) (table 3). 

**Table 3:** Mean lipid profile in elderly with iron deficiency anemia (117) and the control (without iron deficiency anemia) (1176)

**Pvalue**	**Control** **(mean±SD)**	**Iron deficiency anemia** **(mean±SD)**	**Variable**
0.001	158.98±82.15	152.18±108.23	Triglyceride(mg/dL)
0.001	200.54±40.03	193.21±42.18	Cholesterol (mg/dL)
0.036	38.73±4.6	37.03±3.8	HDL (mg/dL)
0.001	134.65±43.21	129.19±44.28	LDL (mg/dL)

**Figure 1:**
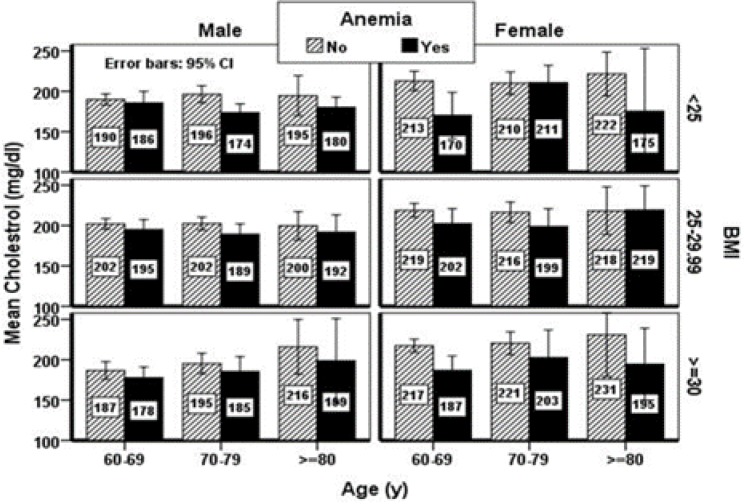
Mean serum level of cholesterol in studied elderly by age, gender, and BMI

The mean serum cholesterol level in anemic and non-anemic elderly of both genders in different groups of body mass index (BMI) is shown in figure 1. Based on this figure, the prevalence of anemia and the mean level of cholesterol are higher among females. Furthermore, the prevalence of anemia slightly increases with the reduction in BMI in both genders. Hemoglobin level showed a significant relationship with triglyceride, cholesterol, and LDL (p=0.001). In addition, TIBC was found to have a significant relationship with cholesterol, HDL, and LDL, while ferritin was only significantly associated with triglyceride (p=0.002). On the other hand, MVC showed a significant relationship with cholesterol and LDL (p=0.002, 0.004). Age was found to be significantly related to cholesterol and triglyceride (p=0.03, 0.001) (table 4).

**Table 4:** Relationship of blood parameters and age with lipid profile

**HDL**	**Triglyceride**	**Cholesterol**	**Hemoglobin**	**Variable**	
-0.004 0.87	0.081 0.001	0.103 0.001	1	Correlation covariance P value	Hemoglobin
0.329 0.001	0.370 0.001	1	0.103 0.001	Correlation covariance P value	Cholesterol
0.111 0.001	1	0.370 0.001	0.081 0.001	Correlation covariance P value	Triglyceride
1	0.111 0.001	0.329 0.001	-0.004 0.87	Correlation covariance P value	HDL
0.198 0.001	0.348 0.001	0.802 0.011	0.091 0.001	Correlation covariance P value	LDL
0.008 0.75	0.019 0.44	0.108 0.001	0.256 0.001	Correlation covariance P value	Serum iron
-0.012 0.63	0.076 0.002	-0.024 0.33	0.015 0.54	Correlation covariance P value	Ferritine
0.058 0.02	-0.029 0.34	-0.093 0.001	-0.203 0.001	Correlation covariance P value	TIBC
-0.028 0.27	-0.022 0.38	0.073 0.004	0.366 0.001	Correlation covariance P value	MCV
-0.018 0.46	-0.124 0.001	-0.052 0.03	-0.160 0.001	Correlation covariance P value	Age

## Discussion

In the present research, the association between triglyceride, cholesterol, HDL, and LDL was studied in the elderly people with anemia and iron deficiency. The results showed that the values of lipid profile including triglyceride and cholesterol in subjects with anemia and IDA were lower than the control. This was also true even after taking into account the effects of age, gender, and BMI.

In the present study, the prevalence of anemia among the ageing individuals was 31%. This figure has been different in various studies. In a study conducted by the Ministry of Health and Medical Education of Iran in December 2004, anemia was observed in 10-11% of the older people (7). In a study conducted in the US, it was reported that nearly 4.7 million Americans suffer from anemia and its prevalence increases with age, as 44.4% of people aged over 85 suffer from it (8). In this study, the prevalence of IDA in the elderly was 9%. About 50% of anemia cases in the world can be attributed to iron deficiency which is responsible for about 841,000 deaths worldwide annually (1). 

A study conducted in Iran in 2001 showed that anemia and iron deficiency occurred in the high percentage of the population in different age groups. Accordingly, about 23% of children under 2, 26% of children aged 6, 23% of male and female adolescents, and 43% of pregnant women suffer from iron deficiency (7). The results of the present study confirmed the findings of Au (20) who reported some degree of association of anemia with lower serum levels of cholesterol. El-Hazmi*et al*. (21) reported that serum level of cholesterol in patients with sickle cell anemia is significantly lower than the control with normal hemoglobin. In the study conducted by Mitrache *et al*. (22), anemic patients showed a significantly lower level of serum cholesterol. In the present study, serum level of cholesterol and triglyceride in anemic group was significantly lower than non-anemic group. Ohira *et al*. (23) found that the serum level of cholesterol increases following the increased hemoglobin through blood transfusion. They argued that the amount of red blood cells probably affects cholesterol synthesis or its displacement from tissue to plasma. 

The relationship between hypercholesterolemia and anemia and also the effect of anemia on atherosclerosis were reported in some studies (24-26). In a study conducted by Ohira*et al*. (28), no significant relationship existed between triglyceride and hemoglobin. By contrast, Choi *et al*. (27) observed a significant relationship between triglyceride and hemoglobin, which is consistent with the findings of the present study. In a study carried out by Bunyaratvej *et al*. (28), there was no relationship between MCV and triglyceride, but a significant inverse relationship was noted between MCV and cholesterol. In the present study, MCV showed a significant relationship between cholesterol and LDL in all subjects, but there was no significant relationship between MCV and triglyceride. 

Choi *et al*. (29) reported no significant relationship between MVC and cholesterol among the elderly. Changes in serum level of cholesterol during iron reduction have been different in many studies conducted on animals. The results of the present study were consistent with the findings of Bristow-Craig *et al*. (18) who showed that consumption of high-iron diet in mice is followed by the elevation of serum cholesterol level. These observations suggest that changes in serum cholesterol level is associated with age, gender, diet, and animal model studies.

By contrast, Stangle and Kirchgessner (30) reported that hypertriglyceridemia in mice is associated with low-iron diets. Guthrie et al. (31) and Amine et al. (32) mentioned that anemia is caused by low-iron diets, it leads to hyperlipidemia, which is inconsistent with the findings of the present study.

On the other hand, Ece et al. (33) noted that iron deficiency has no effect on lipid profile. Since low-iron diets cause loss of energy and protein and thereby lead to a hypocaloric diet, it causes hyperlipidemia. Dabbagh et al. (25) showed that high iron increases serum level of HDL in mice. Nevertheless, in another study, they did not confirm the hypothesis that increased iron reserves increase the risk of coronary artery disease. In the present study, there was significant difference between iron deficiency anemia group and the control in terms of HDL level. One of the main strengths of the present study was the considerable number of subjects and its major weakness was its cross-sectional method which lowers the power of assessment of causal relationship. One of the constraints of this research was the non-delivery or not mentioning the name of drugs related to anemia and hyperlipidemia by the subjects.

In conclusion, the results of the present study showed that the values of lipid profile including triglyceride and cholesterol in subjects with anemia and iron deficiency anemia were lower than the control, which is consistent with the findings of some other studies. Nonetheless, to identify the possible mechanisms, further studies are recommended to be carried out.
